# Zinc finger transcription factor ecotropic viral integration site 1 is induced by all-*trans* retinoic acid (ATRA) and acts as a dual modulator of the ATRA response

**DOI:** 10.1111/j.1742-4658.2009.07398.x

**Published:** 2009-11

**Authors:** Sonja C Bingemann, Torsten A Konrad, Rotraud Wieser

**Affiliations:** 1 Department of Medical Genetics, Medical University of ViennaAustria; 2 Department of Medicine I, Medical University of ViennaAustria

**Keywords:** all-*trans* retinoic acid, EVI1, feedback inhibition, RARE, transcription regulation

## Abstract

*Ecotropic viral integration site 1* (*EVI1*) plays important roles in leukaemia and development, and its expression is temporally and spatially highly restricted during the latter process. Nevertheless, the only physiological agent that to date has been shown to regulate transcription of this gene in mammalian cells is all-*trans* retinoic acid. Here we describe the identification of a retinoic acid response element that was located in the most distal of several alternative first exons of the human *EVI1* gene and was constitutively bound by canonical retinoid receptors in NTERA-2 teratocarcinoma cells. Furthermore, it was the target of negative feedback by EVI1 on the induction of its own promoter by retinoic acid. This process required a previously described transcription repression domain of EVI1. Extending its role as a modulator of the retinoic acid response, EVI1 had the opposite effect on the *RARβ* retinoic acid response element, whose induction by all-*trans* retinoic acid it enhanced through a mechanism that involved almost all of its known functional domains. Augmentation of the retinoic acid response by EVI1 was also observed for the endogenous *RARβ* gene. Thus, we have established EVI1 as a novel type of modulator of the retinoic acid response, which can both enhance and repress induction by this agent in a promoter-specific manner.

## Introduction

*Ecotropic viral integration site 1* (*EVI1*) is an evolutionarily conserved gene with important roles both in malignant diseases and in normal development [1]. Its homozygous disruption in mice led to multiple malformations, including small or missing limb buds, defects in the development of the central and peripheral nervous systems and an immature heart. *Evi1*−/− mice died before 11.5 days postcoitum due to heart failure and massive haemorrhaging [2]. EVI1 is thought to exert its biological effects mainly by acting as a regulator of gene transcription [1]. It codes for a 1051 amino acid nuclear protein with two sets of zinc finger domains that contain seven and three zinc finger motifs, respectively, are able to bind to DNA independently of each other, and are separated from each other by an intervening region (IR) and a transcription repression domain (RD). At the C-terminus of EVI1, an acidic region (AR) involved in transcription activation is present [1]. EVI1 interacts with transcriptional coactivators and corepressors, and is able to both activate and repress gene transcription [3–7]. However, so far only very few direct EVI1 target genes have been described [7,8] and EVI1 may exert its effects in part by modifying the activity of other sequence-specific transcription factors [9–12].

**Fig. 1:**
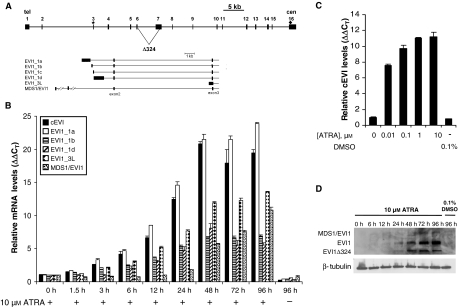
Induction of different *EVI1* mRNA and protein variants in response to ATRA in NTERA-2 cells. (A) Schematic of the human *EVI1* gene and its mRNA splice and 5′-end variants. Boxes, exons; lines, introns. The upper panel shows the entire *EVI1* gene, with alternative splicing producing the Δ324 mRNA variant indicated by triangular lines. Asterisk and diamond, positions of the start codon in exon 3 and of the stop codon in exon 16, respectively. In the lower panel, only the 5′-end of the gene with several alternative first exons is shown. The alternative first exon contained within EVI1_1a is exon 1a, etc. Reproduced with permission from [1]. (B) NTERA-2 cells were treated with 10 μm ATRA or an equivalent amount of dimethylsulfoxide for the indicated periods of time. RNA was extracted and reverse transcribed, and the mRNA levels of cEVI1 (i.e. the sum of all *EVI1* transcripts, amplified using forward and reverse primers located in exons 7 and 8/9, respectively; black columns), EVI1_1a (white columns), EVI1_1b (horizontally striped columns), EVI1_1d (diagonally striped columns), EVI1_3L (checked columns) and MDS1/EVI1 (stippled columns) were assayed by RTQ-RT-PCR. The expression of each *EVI1* mRNA variant relative to the housekeeping gene *cyclophilinD* and the 0 h time point, as well as standard deviations between replicate measurements, were calculated using the ΔΔC_T_ method [45]. Please note that because the ΔΔC_T_ method relates expression values to the 0 h time point for each amplicon, only induction factors, but not expression levels, can be compared between different amplicons. (C) NTERA-2 cells were treated with the indicated amounts of ATRA or dimethylsulfoxide for 15 h. RTQ-RT-PCR for cEVI1 was performed as described above. (D) NTERA-2 cells were treated with 10 μm ATRA or an equivalent amount of dimethylsulfoxide for the indicated periods of time. Proteins were extracted and subjected to immunoblot analysis using an antibody against EVI1, or a β-tubulin antibody as a loading control. The EVI1 antibody detected three bands whose molecular masses corresponded to those of MDS1/EVI1 (∼ 170 kDa), EVI1 (∼ 140 kDa) and of the protein product of an internal splice variant of EVI1, Δ324 (∼ 100 kDa).

The human *EVI1* gene has several different first exons, whose alternative use gives rise to the transcript variants EVI1_1a, EVI1_1b, EVI1_1c, EVI1_1d, and EVI1_3L (Fig. 1A) [1,13–15]. The transcriptional start sites and the putative regulatory regions of these mRNA variants are located in close vicinity to each other, and they are all predicted to be translated into the same 1051 amino acid protein [14]. In contrast, the mRNA variant MDS1/EVI1 gives rise to an EVI1 protein with an extension of 188 amino acids at its N-terminus [16,17], which, at least in some experimental systems, acted differently from, and even in an opposite manner to, the 1051 amino acid EVI1 protein [3,18–20]. The MDS1/EVI1 transcript consists of parts of the mRNA from the *MDS1* gene, joined to the second exon of the EVI1 mRNA [16,17]. The first exon of *MDS1*, and therefore also its presumptive regulatory regions, are located more than 500 kb upstream of *EVI1* exon 1a. Despite this, the expression patterns of MDS1/EVI1 resembled those of the other EVI1 mRNA variants in almost all investigated human and murine tissues [14,17].

Although *EVI1* was expressed in a temporally and spatially highly restricted manner during mammalian development [2,21], the only physiological agent that to date has been identified as potentially contributing to this regulation is all-*trans* retinoic acid (ATRA) [14,22,23]. Interestingly, mice with a homozygous disruption of the *retinaldehyde dehydrogenase-2* gene, which is responsible for embryonic retinoic acid (RA) synthesis, die on the same day of embryonic development as *Evi1*−/− mice, with a partially overlapping phenotype [2,24]. Like *EVI1* [2,22], ATRA also regulates many aspects of neuronal differentiation [25]. Together, these observations suggest that *EVI1* may be involved in mediating some of the biological effects of ATRA.

RA regulates gene expression by binding to nuclear receptors that directly act as transcription factors at the promoters of target genes. Two classes of retinoid receptors have been described: retinoic acid receptors (RARs) and retinoid X receptors (RXRs). Each receptor class has three paralogous members in human cells, designated α, β and γ [26,27]. Gene regulation by RA is usually mediated by heterodimers between one member of each of the RAR and the RXR families. These bind to retinoic acid response elements (RAREs) that consist of two direct repeats (DR) of the consensus sequence PuG(G/T)TCA, separated by one, two or, most commonly, five spacer nucleotides, and accordingly termed DR1, DR2 or DR5 elements. RAR and RXR bind to their cognate response elements in a constitutive manner. In the absence of RA, they repress target gene transcription, whereas ligand binding activates target promoters by inducing conformational changes in the receptors that lead to replacement of corepressors by coactivators [26,27]. Numerous genes have been reported to be regulated by retinoid receptors in a direct manner [28]. In addition, RARs and RXRs have featured both synergistic and antagonistic interactions with other sequence-specific transcription factors [26].

*EVI1* was induced by ATRA in several human and murine cell lines, and this induction was at least in part due to an increased rate of transcription [14,22,23]. Nevertheless, so far no RARE has been found in the regulatory regions of the *EVI1* gene [22]. Here we describe the identification of a functional RARE that is located in exon 1a of the human *EVI1* gene, that binds to retinoid receptors in a constitutive manner, and whose induction by ATRA is inhibited by EVI1. Furthermore, we show that EVI1 augments the induction of the *RARβ* gene by ATRA.

## Results

### Induction of different EVI1 mRNA and protein variants by ATRA in NTERA-2 cells

To characterize the regulation of the *EVI1* gene by ATRA, NTERA-2 cells were incubated with 10 μm of this agent for various periods of time, and the expression of *EVI1* and its mRNA 5′-end variants (except for EVI1_1c, whose levels are too low for reliable detection [13,14]) was measured by real-time quantitative RT-PCR (RTQ-RT-PCR). EVI1_1a, EVI1_1b, EVI1_1d and EVI1_3L (Fig. 1A), as well as the cEVI1 amplicon (which extends from the 3′-end of exon 7 to the 5′-end of exon 9, and thus represents all *EVI1* transcript variants, Fig. 1A) were induced by ATRA to varying extents, but with similar kinetics: their levels were elevated as early as 3 h after the addition of ATRA, continued to rise until after 48 h, and then remained constant until at least 96 h after ATRA addition (Fig. 1B). The MDS1/EVI1 mRNA, in contrast, began to slightly increase in abundance 24 h after the addition of ATRA, was distinctly induced at 48 h and further accumulated up to the 96 h time point (Fig. 1B).

In a dose–response experiment, cEVI1 was strongly induced by as little as 10 nm ATRA and reached maximal levels at 1 μm ATRA (Fig. 1C).

Immunoblot analyses of extracts prepared from NTERA-2 cells treated with 10 μm ATRA for various periods of time revealed three bands specifically recognized by an EVI1 antibody (Fig. 1D). The molecular masses of these bands were consistent with those reported for MDS1/EVI1 (∼ 170 kDa), EVI1 (∼ 140 kDa) and the protein product of an alternatively spliced EVI1 mRNA, Δ324 (∼ 100 kDa) [16,29]. The 140 kDa band, which gave the strongest signal, began to appear 12–24 h after the addition of ATRA and increased in intensity up to the 96 h time point. The 100 kDa band was considerably fainter, but exhibited similar induction kinetics. The 170 kDa band was the faintest, and was detectable only at the 72 and 96 h time points (Fig. 1D). The immunoblot and RTQ-RT-PCR analyses consistently showed that EVI1 was induced strongly and rapidly upon incubation of NTERA-2 cells with ATRA, whereas MDS1/EVI1 was upregulated with delayed kinetics.

### An inverted DR5 RARE in *EVI1* exon 1a confers ATRA responsiveness to the *EVI1* promoter and binds canonical retinoid receptors in a constitutive manner

**Fig. 2:**
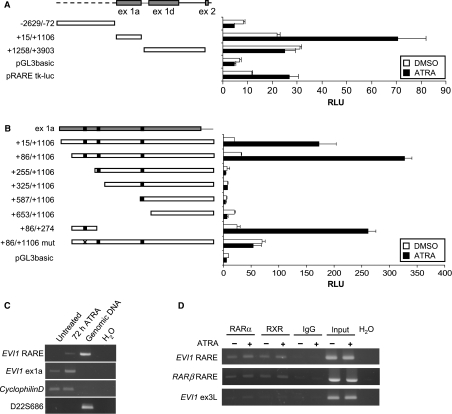
A functional RARE is located in exon 1a of the human *EVI1* gene. (A) Schematic of the genomic region at the 5′-end of the human *EVI1* gene and of the fragments cloned into pGL3basic, and luciferase reporter assays. Stippled line, *EVI1* upstream region; grey boxes, exons (exons 1b and 1c are too small to be depicted in this schematic); solid lines, introns. The positions of cloned fragments are indicated relative to the transcriptional start site of EVI1_1a (GenBank accession no. BX640908). pRARE-tk-luc contains two copies of the human *RARβ* RARE and the *tk* minimal promoter in pGL2, and was used as a positive control for the ATRA response. For luciferase assays, the indicated reporter plasmids were transfected into NTERA-2 cells, together with the renilla luciferase plasmid pRL; 10 μm ATRA or an equivalent amount of dimethylsulfoxide was added 1 day after transfection. Another ∼ 24 h later, cells were lysed and luciferase activities were measured. Relative luciferase units (RLU) were derived by normalizing firefly to renilla luciferase activities. Error bars represent the standard deviations between duplicate measurements. White bars, dimethylsulfoxide; black bars, ATRA. (B) Schematic of EVI1(+15/+1106)/pGL3 and its derivative constructs, and luciferase reporter assays. Grey box, exon 1a; black boxes, predicted RAREs. In EVI1(+86/+1106)mut/pGL3, the six nucelotides comprising the second half-site of the *EVI1* RARE were mutated (indicated by a cross). Luciferase reporter gene assays were performed as in (A). (C) RT-PCR confirming that the RARE of the *EVI1* gene is located within its transcribed region. NTERA-2 cells were incubated with ATRA for 0 or 72 h. RNA was extracted, treated with DNaseI, reverse transcribed and amplified using primers flanking the *EVI1* RARE (EVI1_RARE-F, EVI1_RARE-R; Table S1B), an intron-spanning primer pair located in a more proximal region of EVI1_1a (EVI1_1a-fwd, EVI1_1a-rev; Table S1A) and a primer pair for the housekeeping gene *cyclophilinD* (cycD-F, cycD-R; Table S1A). The effectiveness of the DNaseI treatment was verified with primers for the microsatellite marker D22S686 (D22S686-F, D22S686-R; Table S1B). (D) ChIP was performed on NTERA-2 cells treated with ATRA or dimethylsulfoxide for 24 h using an antibody specific to RARα, a pan-RXR antibody, or unspecific rabbit IgG as a negative control. Immunoprecipitated chromatin and input DNA as a positive control were amplified with primers flanking the *EVI1* RARE (EVI1_RARE-F, EVI1_RARE-R; Table S1B), primers for the *RARβ* RARE (RARβ_RARE-F, RARβ_RARE-R; Table S1B) or with negative control primers located in *EVI1* exon 3L (EVI1_ex3L-F; EVI1_ex3L-R; Table S1B).

To search for a RARE in the putative regulatory regions of the human *EVI1* gene, three fragments that together covered most of the genomic region from positions −2.6 to +3.9 kb relative to the 5′-end of *EVI1* exon 1a were cloned into the luciferase reporter vectors pGL3basic and pGL3promoter (Fig. 2A). Only EVI1(+15/+1106)/pGL3, whose insert largely corresponds to *EVI1* exon 1a, but not EVI1(−2629/−72)/pGL3 or EVI1(+1258/+3903)/pGL3, responded to ATRA in reporter gene assays (Fig. 2A, and data not shown). Because pGL3basic- and pGL3promoter-based constructs yielded comparable results, subsequent experiments were performed only with pGL3basic. Deletion of the first 240 nucleotides of EVI1(+15/+1106)/pGL3 completely eliminated the ATRA response (Fig. 2B), suggesting that it was mediated by the inverted DR5 RARE consensus sequence CGACCTTTTTGTGACCT present between nucleotide positions +182 and +198 relative to the beginning of exon 1a. Indeed, EVI1(+86/+274)/pGL3 was still responsive to ATRA. The importance of the RARE consensus sequence in conferring ATRA responsiveness to the *EVI1* promoter was corroborated through mutation of the six residues comprising its second half-site, which yielded EVI1(+86/+1106)mut/pGL3. This construct did not exhibit any ATRA response (Fig. 2B). However, its basal activity was elevated as compared with that of the corresponding wild-type construct (Fig. 2B), probably due to the loss of repression by unliganded RARs and RXRs [27].

To confirm that the *EVI1* RARE was located within the transcribed region of the *EVI1* gene, RT-PCR with primers surrounding the RARE (Table S1B) was performed on DNAse-treated RNA from NTERA-2 cells that had been incubated with ATRA or vehicle for 72 h. The RARE amplicon was elevated in response to ATRA (Fig. 2C), demonstrating that ATRA-induced transcription of the *EVI1* gene indeed initiates in part or entirely upstream of its RARE.

Chromatin immunoprecipitation (ChIP) experiments revealed that RARα and RXR were associated with the *EVI1* RARE both in the absence and presence of ATRA (Fig. 2D). Confirming the specificity of these interactions, only faint bands were observed when negative control immunoprecipitates or primers were used (Fig. 2D). Similar results were obtained for the RARE of the *RARβ* gene promoter, which was employed as a positive control (Fig. 2D). In summary, the first exon of the *EVI1* gene contains a consensus RARE motif that is bound by canonical RARs and RXRs, which repress it in the absence and activate it in the presence of ligand.

### ATRA regulates the *MDS1/EVI1* promoter neither directly, nor through EVI1

**Fig. 3:**
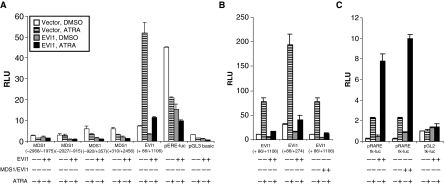
EVI1 does not mediate ATRA regulation of the *MDS1/EVI1* promoter, but decreases the ATRA response of its own RARE and enhances that of the *RARβ* RARE. (A) Four genomic fragments, whose positions are indicated relative to the transcriptional start site of the *MDS1/EVI1* gene (assumed to be identical to that of the *MDS1* gene; GenBank accession no. NM_004991), were cloned into the luciferase reporter vector pGL3basic. NTERA-2 cells were cotransfected with these reporter constructs, HA-EVI1/pEFzeo or empty vector, and the renilla luciferase vector pRL-SV40. EVI1(+86/+1106)/pGL3 served as a positive control for the ATRA response; pEREluc, which contains two copies of an EVI1 DNA binding site, as a positive control for the effects of EVI1; and empty pGL3basic as a negative control. ATRA or dimethylsulfoxide was added 1 day after transfection, and cells were lysed and luciferase activities determined another ∼ 24 h later. Relative luciferase units (RLU) were derived by normalizing firefly to renilla luciferase activities. Error bars represent the standard deviations between duplicate measurements. (B, C) Luciferase assays after transfection of NTERA-2 cells with the indicated reporter plasmids and the expression vectors HA-EVI1/pEFzeo or HA-MDS1/EVI1/pEFzeo were performed as described in (A). pRARE-tk-luc contains two copies of the RARE of the human *RARβ* gene promoter and the *tk* minimal promoter in pGL2; pGL2-tk-luc contains only the *tk* minimal promoter in pGL2.

Because ATRA induced the MDS1/EVI1 mRNA only slowly (Fig. 1B), its action on the *MDS1/EVI1* promoter seemed unlikely to be direct. Indeed, none of four overlapping fragments, which together covered the genomic region from position −3.0 to +2.5 kb relative to the transcriptional start site of *MDS1/EVI1*, conferred responsiveness to treatment with ATRA for 24 or 48 h to a luciferase reporter (Fig. 3A, and data not shown). We therefore asked whether ATRA might regulate the slowly responding *MDS1/EVI1* promoter in an indirect manner through the rapidly induced EVI1 protein. However, none of the *MDS1/EVI1* promoter fragments was induced by exogenously expressed EVI1, either in the absence or in the presence of ATRA (Fig. 3A). Therefore, even though ATRA clearly induces *MDS1/EVI1*, the mechanism through which it does so remains unclear at present.

pERE/luc served as a positive control for the effects of EVI1 in the reporter assays, and showed the expected decrease in luciferase activity upon ectopic expression of EVI1 (Fig. 3A). Notably, ATRA also reduced the activity of pERE/luc (Fig. 3A), probably through its ability to upregulate the expression of endogenous EVI1. This supports the assumption that EVI1 protein induced by ATRA in NTERA-2 cells is functional as a transcriptional regulator. Interestingly, ATRA induction of EVI1(+86/+1106)/pGL3, which was included in the experiment as a positive control for the ATRA response, was strongly reduced in the presence of exogenously expressed EVI1 (Fig. 3A), suggesting negative feedback by EVI1 on its own promoter.

### Both EVI1 and MDS1/EVI1 counteract the ATRA response of the *EVI1* promoter, but enhance that of the *RARβ* RARE

As with EVI1(+86/+1106)/pGL3, the ATRA response of EVI1(+86/+274)/pGL3 was strongly reduced by exogenously expressed EVI1 (Fig. 3B). This indicated that EVI1 may interfere directly with the action of RAR, RXR and/or closely associated proteins at its own promoter. We next asked whether the repressive effects of EVI1 would extend to other ATRA responsive genes. Surprisingly, ATRA induction of pRARE-tk-luc, which contains two copies of the RARE that is present in the human *RARβ* gene promoter [30], was not diminished, but rather enhanced, by EVI1 (Fig. 3C). Like EVI1, MDS1/EVI1 also counteracted ATRA induction of the *EVI1* promoter and enhanced that of the *RARβ* RARE (Fig. 3B,C).

### Protein domains involved in modulation of the ATRA response by EVI1

**Fig. 4:**
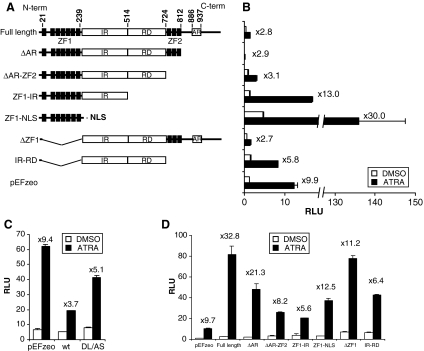
Protein domains involved in negative and positive modulation of the ATRA response by EVI1. (A) Schematic of the *EVI1* deletion constructs. Black boxes, zinc finger motifs; ZF1, ZF2, zinc finger domains 1 and 2, respectively; IR, intervening region; RD, repression domain; AR, acidic region. Amino acid positions delimiting these domains are indicated. All constructs are based on the pEFzeo vector backbone, and contain an N-terminal HA epitope tag. The SV40 large T antigen NLS was engineered onto the ZF1 construct; all other constructs include a predicted NLS contained in the IR. (B–D) Luciferase assays using the indicated *EVI1* deletion constructs along with the reporter vectors EVI1(+86/+1106)/pGL3 (B, C) or pRARE-tk-luc (D) were performed as described in Fig. 3. wt, wild-type EVI1; DL/AS, CtBP binding site mutant. White bars, dimethylsulfoxide; black bars, ATRA. Fold induction by ATRA in the presence of each construct is indicated. The effects of all mutations were highly reproducible; only deletion of the AR diminished the activity of EVI1 on pRARE-tk-luc more modestly in an independent experiment.

In order to identify regions in the EVI1 protein that contribute to positive and negative modulation of the ATRA response, a series of *EVI1* deletion constructs was prepared (Fig. 4A). All constructs had an N-terminal hemagglutinin (HA) epitope tag, and either retained a predicted nuclear localization sequence (NLS) in the IR, or were engineered to contain an NLS. The expression and nuclear location of the truncated proteins was confirmed by immunoblot and indirect immunofluorescence analyses (Fig. S1).

Deletion of the first zinc finger domain (ZF1), of the C-terminal AR, or of the AR and the second zinc finger domain (ZF2) (Fig. 4A, B: ΔZF1, ΔAR and ΔAR-ZF2, respectively) did not affect the repressive effects of EVI1 on the ATRA response of EVI1(+86/+1106)/pGL3 (Fig. 4B). However, a mutant with a C-terminal deletion that removed the RD in addition to the AR and the ZF2 (Fig. 4A, B: ZF1-IR) was inactive. Further deletion of the IR to yield a construct consisting only of the ZF1 (Fig. 4A, B: ZF1-NLS) even enhanced the ATRA response of the *EVI1* promoter (Fig. 4B). A truncated EVI1 protein comprising only the IR and the RD (Fig. 4A, B: IR-RD) still had repressive activity, albeit at a reduced level (Fig. 4B). The RD was thus clearly important for the negative feedback by EVI1 on its own promoter. It contains two closely spaced binding sites for the corepressor protein C-terminal binding protein (CtBP), the more C-terminal one of which was reported to be functionally more important [4,5,31]. Mutation of this site on the background of the full-length EVI1 protein only moderately decreased the ability of EVI1 to counteract the ATRA response, suggesting that CtBP was not primarily responsible for transcriptional repression by EVI1 in this context (Fig. 4C).

To test which domains of EVI1 were involved in enhancing the ATRA response of the *RARβ* RARE, similar experiments were performed using the pRARE-tk-luc reporter plasmid. Deletion of the AR (Fig. 4A, D: ΔAR) had only a modest effect on the enhancement of the ATRA response by EVI1, but deletion of both the AR and the ZF2 (Fig. 4A, D: ΔAR-ZF2) eliminated it (Fig. 4D). Likewise, an EVI1 protein devoid of the ZF1 (Fig. 4A, D: ΔZF1), or constructs encoding only the ZF1 (Fig. 4A, D: ZF1-NLS), the ZF1 and IR (Fig. 4A, D: ZF1-IR), or the IR and RD (Fig. 4A, D: IR-RD), were incompetent to increase the ATRA response of pRARE-tk-luc (Fig. 4D). Taken together, several or all EVI1 protein domains contributed to the enhancement of the ATRA response by this transcription factor.

### EVI1 increases the ATRA response of the endogenous *RARβ* gene in U937T cells

**Fig. 5:**
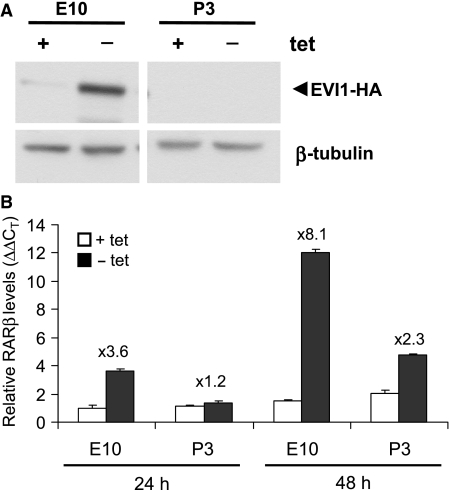
EVI1 enhances the ATRA response of the endogenous *RARβ* gene in U937T_EVI1-HA cells. (A) Immunoblot analysis demonstrating induction of EVI1-HA in U937T_EVI1-HA E10 cells (E10) 48 h after removal of tetracycline (tet) from the culture media. U937T cells transfected with empty plasmid (U937T_EP P3 cells; P3) were used as a negative control. EVI1-HA was detected with an HA antibody; hybridization with an antibody against β-tubulin was used as a loading control. (B) U937T_EVI1-HA E10 cells (E10) and U937T_EP P3 cells (P3) were maintained in the presence or absence of tetracycline and of ATRA for 24 and 48 h. RARβ mRNA levels were determined by RTQ-RT-PCR and related to those of the housekeeping gene *cyclophilinD* using the ΔΔC_T_ method [45]. Because *RARβ* expression was undetectable in the absence of ATRA, the corresponding data are not shown. White and black columns, cells cultured in the presence or absence of tetracycline, respectively.

Finally, we asked whether EVI1 would be able to modulate induction of an endogenous gene by ATRA. In NTERA-2 cells, endogenous EVI1 was likely to obscure the effects of experimentally expressed EVI1. To circumvent this problem, the cell line U937T_EVI1-HA E10, a U937 derivative that does not express its own *EVI1* gene, but has been engineered to express exogenous EVI1 in a tetracycline repressible manner (Fig. 5A) [32], was used. U937T_EP P3 cells, which contain the corresponding empty plasmid instead of the *EVI1* expression plasmid, served as a negative control. In U937T_EVI1-HA E10 cells, the RARβ mRNA was undetectable by RTQ-RT-PCR in the absence of ATRA, but was strongly upregulated by this agent. Induction of EVI1 by removal of tetracycline led to an enhancement of this response (Fig. 5B), demonstrating that EVI1 was indeed able to modulate the ATRA response of an endogenous gene.

## Discussion

Expression of the *EVI1* gene is highly regulated during normal development [2,21,33] and is deregulated in certain types of cancer [13,15,34–36]. Nevertheless, little is known about the control of its transcription. In the present report, we have confirmed induction of the human *EVI1* gene by ATRA, and demonstrated that a classical inverted DR5 RARE is located within its transcribed region, near the beginning of exon 1a. This element was necessary and sufficient to confer ATRA responsiveness to a luciferase reporter gene. Like other RAREs [26,27], it was bound by RAR and RXR in a constitutive manner, and repressed in the absence and activated in the presence of ligand. *In silico* analysis also predicted a DR2 RARE in exon 1 of the *MDS1/EVI1* gene. However, neither this element nor any other sequences in a 5.5 kb region surrounding the transcriptional start site of *MDS1/EVI1* were able to confer ATRA responsiveness to a luciferase reporter gene. Also, *MDS1/EVI1* was not regulated by ATRA indirectly through the rapidly induced EVI1 protein. It remains possible, however, that regulatory elements outside the region investigated in this study do indeed confer direct responsiveness to ATRA and/or EVI1 to the *MDS1/EVI1* promoter.

Interestingly, we found that the *EVI1* RARE not only mediated transcriptional induction in response to ATRA, but in addition conferred negative feedback by EVI1 on its own promoter. On the other hand, EVI1 enhanced the ATRA response of a reporter gene driven by the *RARβ* RARE, as well as that of the endogenous *RARβ* gene. This not only argues against a nonspecific squelching effect as an explanation for the negative feedback of EVI1 on its own promoter, but also establishes EVI1 as a dual regulator of the ATRA response, which is able to repress or enhance gene induction by this agent in a promoter-specific manner. Notably, *in silico* analysis using tfsearch (http://www.cbrc.jp/research/db/TFSEARCH.html) did not reveal any EVI1 consensus DNA binding sites overlapping with, or located in the vicinity of, the *EVI1* or the *RARβ* RAREs. Accordingly, ChIP experiments using either an EVI1 or an HA antibody failed to reveal an interaction between the EVI1 protein and the *EVI1* or *RARβ* RAREs in native or transfected NTERA-2 cells, or in U937T_EVI1-HA cells (data not shown). This may be due to technical problems related to, for example, antibody quality, epitope accessibility or the positioning of side chains reactive with cross-linking reagents. Alternatively, modulation of the ATRA response by EVI1 may not involve interactions between EVI1 and DNA. Instead, the induction of *EVI1* and *RARβ* by ATRA may require different cofactors, and EVI1 may differentially interact with, and affect the activity of, these cofactors in the nucleoplasm.

Not surprisingly, the positive and negative effects of EVI1 on the ATRA response were mediated through different protein domains. For the inhibitory effects of EVI1 on the induction of its own promoter by ATRA, the region between the two zinc finger domains was both necessary and sufficient. This region consists of a previously established RD [4,5,37] and an IR, which may also be able to mediate transcriptional repression [38]. Even though expression of the isolated IR+RD considerably diminished the ATRA response of the *EVI1* promoter, full repression required the additional presence of either N-terminally or C-terminally adjacent domains. These may contribute in a partially redundant manner to pertinent interactions between EVI1 and other proteins, and/or be required to maintain the IR and RD in the conformation they adopt in the context of the full-length protein. A point mutation (DL/AS) disrupting the functionally more important of two consensus binding sites for the corepressor CtBP in the EVI1 RD [4,5] reduced the negative modulation of the ATRA response by EVI1. However, EVI1 DL/AS also exhibited a diminished ability to enhance the ATRA response of the *RARβ* RARE (data not shown), an activity unlikely to be mediated by a corepressor. This suggested that the DL/AS mutation may not only affect CtBP binding, but may also alter the structure of EVI1 and/or its interactions with other proteins. Other corepressors shown to functionally interact with EVI1, such as histone deacetylase 1 [6] or the histone methyl transferase SUV39H1 [39,40], may therefore contribute to, or mediate, repression of the ATRA response by EVI1. As for the ability of EVI1 to enhance ATRA induction of the *RARβ* gene, several of its protein domains appeared to take part in it. Deletion of the AR, which had previously been implicated in transcriptional activation by EVI1 [41,42], only moderately reduced the activating effect of EVI1 on the *RARβ* RARE. However, further deletion of the ZF2, or deletion of only the ZF1, abolished this activity, which therefore appears to require multiple and complex macromolecular interactions.

It remains to be determined whether EVI1 modulates more of the several hundred reported ATRA responsive genes [28] in a similar manner as the *EVI1* or *RARβ* genes. EVI1 probably indirectly affects the expression of at least some of them through the increased levels of RARβ it contributes to. Because ATRA-induced transcription of the *EVI1* gene itself would probably also be augmented through this mechanism, a potentially detrimental self-amplifying feed-forward loop could ensue. The negative feedback of EVI1 on the ATRA induction of its own promoter plausibly serves to prevent such a self-enhancing regulatory loop.

Our results also shed new light on previous observations that ectopic expression of *EVI1*, possibly in concert with low amounts of ATRA naturally present in sera used for cell culture, mimicked neuronal differentiation of P19 cells in response to exogenously added ATRA [22], and that targeted disruption of the *retinaldehyde dehydrogenase-2* and *Evi1* genes in mice caused partially overlapping phenotypes [2,24]: EVI1 appears to act both as a downstream target of the ATRA response and as an upstream regulator of it. It may therefore contribute to the biological effects of ATRA in a dual manner: first, by activating or repressing its own, direct target genes; and second, by modulating the expression of retinoid receptor-regulated genes. Another exciting possibility raised by our results is that *EVI1* overexpressing leukaemias may exhibit increased sensitivity to ATRA-induced differentiation. If this presently speculative assumption can be substantiated by further experiments, new treatment possibilities may emerge for myeloid leukaemia with *EVI1* overexpression, a disease entity that is notoriously resistant to currently used therapeutic regimens [15,36,43].

## Materials and methods

### Cell culture

The human teratocarcinoma cell line NTERA-2 (ACC 527) was obtained from the German Collection of Microorganisms and Cell Cultures, Braunschweig, Germany. It was cultivated in Dulbecco’s modified Eagle’s medium (Invitrogen, Carlsbad, CA, USA) supplemented with 10% fetal bovine serum (Invitrogen) and 5% horse serum (Invitrogen) in a humidified 37 °C incubator at 5% CO_2_. U937T cells, kindly provided by G. Grosveld, St Jude Hospital, Memphis, TN, USA, had been derived from U937 human histiocytic lymphoma cells by stable transfection with a construct driving tetracycline regulable expression of the tetVP16 fusion protein [44]. Transfection with an expression plasmid coding for a tetracycline regulable, HA epitope tagged EVI1 protein or with empty plasmid as a control yielded U937T_EVI1-HA E10 and U937T_EP P3 cells, respectively [32]. These were maintained at 37 °C and 5% CO_2_ in RPMI 1640 medium (Invitrogen) supplemented with 10% fetal bovine serum, 500 μg·mL^−1^ hygromycin (PAA, Pasching, Austria), 0.5 μg·mL^−1^ puromycin (Sigma, St Louis, MO, USA) and 1 μg·mL^−1^ tetracycline (Serva, Heidelberg, Germany). To induce the expression of EVI1-HA, cells were washed three times with phosphate-buffered saline and resuspended in growth media without tetracycline.

ATRA stock solutions contained 10 mm ATRA in dimethylsulfoxide. Unless indicated otherwise, ATRA was used at final concentrations of 10 and 1 μm for NTERA-2 cells and U937T derivative cell lines, respectively.

### RNA isolation, cDNA synthesis and RTQ-RT-PCR

Total RNA was extracted using Trizol (Invitrogen), treated with RNase-free DNaseI (Invitrogen), and reverse transcribed using random hexamer primers (Invitrogen) and M-MLV reverse transcriptase (Invitrogen) according to the manufacturer’s instructions. RTQ-RT-PCR was carried out in an ABI Prism 7700 Sequence Detection System (Applied Biosystems, Carlsbad, CA, USA) using the *Power* SYBR® GREEN PCR Master Mix (Applied Biosystems) according to the manufacturer’s instructions. Primers are shown in Table S1A. All primers exhibited optimal amplification efficiencies in serial dilution experiments. Assays for the housekeeping gene *cyclophilinD* were carried out in duplicate; all other assays were carried out in triplicate. Expression values for the genes of interest relative to *cyclophilinD* and to a reference value were determined using the ΔΔC_T_ method [45].

### Plasmids

*EVI1* promoter fragments −2629/−72, +15/+1106 and +1258/+3903 (relative to the transcription start site of EVI1_1a, GenBank accession no. BX640908) were amplified from BAC clone RP11-33A1 using Dynazyme (Finnzyme, Espoo, Finland; a DNA polymerase with proofreading activity) and primers containing engineered restriction sites. Similarly, *MDS1/EVI1* promoter fragments −2968/−1975, −2027/−915, −920/+357 and +310/+2456 (relative to the transcription start site of *MDS1*, GenBank accession no. NM_004991) were amplified from BAC clone RP11-816J6. PCR products were cloned into pCR® 2.1-TOPO® (Invitrogen) and then transferred into the luciferase reporter plasmid pGL3basic (Promega, Madison, WI, USA). Initial experiments with the *EVI1* promoter fragments were also carried out with analogous constructs in pGL3promoter, but as both types of plasmid yielded similar results, subsequent analyses were restricted to pGL3basic. Deletion subclones of EVI1(+15/+1106)/pGL3 were generated through restriction digestion and religation or through PCR. A mutation that changed the sequence of the second half-site of the *EVI1* RARE from TGACCT to CTTTAG [46] was introduced into EVI1(+86/+1106)/pGL3 using overlap extension PCR. The resulting PCR product was cloned into pCR® 2.1-TOPO® (Invitrogen) and then transferred into pGL3basic. Primers and restriction sites used to generate promoter constructs are available upon request. pRARE-tk-luc contains two copies of the RARE of the human *RARβ* gene promoter [30] and a *tk* minimal promoter in pGL2 (Promega); the corresponding empty control vector pGL2-tk-luc is identical except that it lacks the RAREs. Both plasmids were kindly provided by H. Harant, Ingenetix, Vienna, Austria. pERE/luc consists of two copies of the recognition sequence for the N-terminal zinc finger domain of EVI1, the *tk* minimal promoter and the pGL3basic vector backbone [6].

HA-EVI1/pEFzeo and HA-MDS1/EVI1/pEFzeo contain the human *EVI1* and *MDS1/EVI1* cDNAs, respectively, both with an engineered Kozak consensus for translation initiation and an N-terminal HA epitope tag, under the control of the *eIF1α* promoter [6]. To obtain *EVI1* deletion constructs, internal restriction fragments of HA-EVI1/pEFzeo were replaced by suitable PCR products in a manner that left the Kozak sequence and the HA tag intact. To construct EVI1 ZF1-NLS, a double-stranded oligonucleotide containing a tandem repeat of the SV40 large T antigen NLS [47], followed by a stop codon, and a PCR product covering the EVI1 ZF1 region and part of the NLS were used as templates in a fusion PCR. The resulting ZF1-NLS PCR product was cloned into HA-EVI1/pEFzeo as described above. All other deletion constructs retained a predicted NLS in the IR of EVI1. Primers and restriction sites used to generate EVI1 deletion constructs are available upon request. The human *EVI1* cDNA with the CtBP binding site mutation, DL/AS, and the corresponding wild-type cDNA had been cloned into pME18S, and were kindly provided by M. Kurokawa, Tokyo University, Japan [5].

### Transient transfections and reporter gene assays

One day before transfection, NTERA-2 cells were seeded into 24-well plates at a density of 8 × 10^4^ cells per well. Transfections were carried out using DAC-30 (Eurogentec, Seraing, Belgium; production discontinued) or FuGene6 (Roche, Basel, Switzerland) according to the manufacturers’ instructions, employing 0.6 μg DNA and 2 μL DAC-30, or 0.6 μg DNA and 3 μL FuGene6, per well. Transfection efficiencies were > 25%.

To assess the ATRA responsiveness of the *EVI1* promoter fragments, 540 ng of reporter plasmid were cotransfected with 60 ng of the renilla luciferase plasmid pRL-TK (Promega). To determine the responsiveness of reporter plasmids to EVI1, its deletion derivatives or MDS1/EVI1, 300 ng of reporter plasmid was cotransfected with 300 ng of the respective expression vector and 6 ng of the renilla luciferase plasmid pRL-SV40 (Promega). 10 μm ATRA, or an equivalent amount of dimethylsulfoxide, was added to each well 1 day after transfection. On day two after transfection, the cells were lysed, and firefly and renilla luciferase activities were assayed in an Aureon PhL luminometer (Aureon Biosystems, Vienna, Austria) using the Dual-Luciferase Reporter Assay System (Promega). Firefly luciferase activities were normalized to renilla luciferase activities to adjust for variability in transfection efficiencies. Duplicate samples were assayed for each data point.

### ChIP

NTERA-2 cells were cultivated in 15 cm dishes to ∼ 80% confluence and treated with 10 μm ATRA, or an equivalent amount of dimethylsulfoxide, for 24 h. Proteins were cross-linked to DNA by incubating the cells with 1% formaldehyde (Sigma) for 10 min at 37 °C. After quenching with 125 mm glycine (Sigma) for 5 min at room temperature, the cells were washed with ice-cold phosphate-buffered saline containing a complete protease inhibitor cocktail (Roche), scraped off the dishes and lysed in a buffer containing 50 mm Tris pH 8.1, 10 mm EDTA, 1% SDS and a complete protease inhibitor cocktail (Roche). Chromatin was sheared with eight cycles of 15 s each at 90% duty cycle on a Branson sonifier 450 (Branson, Danbury, CT, USA). Sample concentrations were normalized based on their *A*_280_. Chromatin was diluted 1 : 10 into ChIP buffer (OneDay ChIP kit, Diagenode, Liege, Belgium) and agitated overnight at 4 °C with 8 μg of RARα antibody (sc-551, Santa Cruz, CA, USA), pan-RXR antibody (sc-774, Santa Cruz) or rabbit IgG. Further processing was performed using the OneDay ChIP kit according to the manufacturer’s instructions.

In an aliquot of the sheared chromatin, the cross-link was reversed by overnight incubation at 65 °C with 2% SDS, 100 mm NaHCO_3_, 10 mm dithiothreitol and 200 mm NaCl. After proteinase K digestion, DNA was recovered by phenol/chloroform extraction and ethanol precipitation. Agarose gel electrophoresis showed a DNA smear ranging in size from 100 bp to 2 kb, with maximal intensity at 500 bp. Immunoprecipitated DNA and de-crosslinked (‘input’) DNA as a positive control were subjected to PCR with primers flanking the *EVI1* RARE (EVI1_RARE-F, EVI1_RARE-R; Table S1B), primers flanking the *RARβ* RARE (RARβ_RARE-F, RARβ_RARE-R; Table S1B; [48]), and with negative control primers located in *EVI1* exon 3L (EVI1_ex3L-F, EVI1_ex3L-R; Table S1B).

### Immunoblot analysis

For protein extraction, cells were subjected to repeated freeze–thawing in a buffer containing 20 mm Tris pH 8.0, 100 mm NaCl, 1 mm EDTA, 0.5% NP-40, 0.5 mm phenylmethanesulfonyl fluoride and a complete protease inhibitor cocktail (Roche). Denaturing PAGE, tank blotting onto Bio Trace NT nitrocellulose membranes (Pall Corporation, Port Washington, NY, USA) and antibody hybridizations were performed using standard procedures. Primary antibodies directed against the HA-tag (mouse anti-HA.11 clone 16B12; Covance, Berkeley, CA, USA), EVI1 (rabbit anti-EVI1 C50E12; Cell Signaling Technology, Danvers, MA, USA) or β-tubulin (mouse anti-β-tubulin clone TUB 2.1; Sigma) were used at dilutions of 1 : 10000, 1 : 1000 and 1 : 2500, respectively. Horseradish peroxidase-conjugated goat anti-mouse and goat anti-rabbit secondary IgGs were used at dilutions of 1 : 40000–1 : 100000, and detected using the Super Signal West Femto kit (Pierce, Rockford, IL, USA).

## Abbreviations

AR: acidic region
ATRA: all-*trans* retinoic acid
ChIP: chromatin immunoprecipitation
CtBP: C-terminal binding protein
DR: direct repeat
EVI1: ecotropic viral integration site 1
HA: hemagglutinin
IR: intervening region
NLS: nuclear localization sequence
RA: retinoic acid
RAR: retinoic acid receptor
RARE: retinoic acid response element
RD: repression domain
RTQ-RT-PCR: real-time quantitative RT-PCR
RXR: retinoid X receptor
ZF1: zinc finger domain 1
ZF2: zinc finger domain 2

## Supporting information

The following supplementary material is available:

**Fig. S1.** Expression and nuclear location of protein products derived from *EVI1* deletion constructs.

**Doc. S1.** Immunofluorescence analysis.

**Table S1.** Primers used for RTQ-RT-PCR, RT-PCR, genomic PCR and ChIP PCR.

This supplementary material can be found in the online version of this article.

Please note: As a service to our authors and readers, this journal provides supporting information supplied by the authors. Such materials are peer-reviewed and may be re-organized for online delivery, but are not copy-edited or typeset. Technical support issues arising from supporting information (other than missing files) should be addressed to the authors.
