# Supplementary material for: Zinc finger transcription factor ecotropic viral integration site 1 is induced by all-trans retinoic acid (ATRA) and acts as a dual modulator of the ATRA response
Source: FEBS J. 2009 Nov;276(22):6810–22. doi: 10.1111/j.1742-4658.2009.07398.x (PMC2779989; doi:10.1111/j.1742-4658.2009.07398.x)
Supplement: Supplementary file 1 [file ejb0276-6810-SD1.pdf]

**SUPPLEMENTARY TABLE**

Table S1A: Primers used for RTQ-RT-PCR and for RT-PCR

| Primer name* | Primer sequence† | acc. no‡ | position§ | AT¶ |
|---|---|---|---|---|
| cEVI1-F | ACCCACTCCTTTCTTTATGGACC | NM_005241.2 | 2136–2158 | 60°C |
| cEVI1-R | TGATCAGGCAGTTGGAATTGTG | NM_005241.2 | 2273–2252 | 60°C |
| EVI1_1a-F | TATTGCTGAGTTGAGGCCATAG | NM_001105078.2 | 981-1002 | 60°C |
| EVI1_1a-R | CTTCCAACATCTGGTTGACTGG | NM_001105078.2 | 1074-1053 | 60°C |
| EVI1_1b-F | TGGGTCGTCCGGCCTT | AF487422 | 80-95 | 60°C |
| EVI1_1b-R | GCTTCCAACATCTGGTTGACTG | NM_005241.2 | 117-96 | 60°C |
| EVI1_1d-F | CTTCTTGACTAAAGCCCTTGGA | NM_001105077.2 | 69-90 | 60°C |
| EVI1_1d-R | GTACTTGAGCCAGCTTCCAACA | NM_001105077.2 | 191-170 | 60°C |
| EVI1_3L-F | GGTATCTTAGTGTATATCTTGCCCTTTGT | AF487423 | 263-291 | 60°C |
| EVI1_3L-R | GCGCAATGTCTGCAACTACTCT | NM_005241.2 | 220-199 | 60°C |
| MDS1/EVI1-F | CCAGTTATGGATGGGAGATCTTAGAC | S69002 | 2456-2481 | 60°C |
| MDS1/EVI1-R | CCAGCGAATCTAATGTACTTGAGC | NM_005241.2 | 143-120 | 60°C |
| RARβ-F | AGCAAGCCTCACATGTTTCC | NM_016152 | 1195-1214 | 60°C |
| RARβ-R | ATGAGAGGTGGCATTGATCC | NM_016152 | 1319-1300 | 60°C |
| cycD-F | ATATTGGAAAATGTGGAAGTGAAAGG | NM_005038 | 593-618 | 60°C |
| cycD-R | TCGCCAGAGCCATCTTTTG | NM_005038 | 711-693 | 60°C |
| EVI1_1a-fwd | TATTGCTGAGTTGAGGCCATAG | NM_001105078.2 | 981-1002 | 58°C |
| EVI1_1a-rev | TTGAAAATGCTGAGTGAGGAGT | NM_001105078.2 | 1358-1337 | 58°C |

Table S1B: Primers used for ChIP and genomic PCR

| Primer name* | Primer sequence† | acc. no‡ | position§ | AT¶ |
|---|---|---|---|---|
| D22S686-F | TTGATTACAGAGTGGCTCTGG | G08094 | 77-96 | 58°C |
| D22S686-R | TAAGCCCTGTTAGCACCACT | G08094 | 260-237 | 58°C |
| EVI1_RARE-F | GGGGTACC_CACACCACACTTGTGCTT | NM_001105078.2 | 15-32 | 58°C |
| EVI1_RARE-R | GAAGATCT_AAAGTGACAGCAGCCTCCTC | NM_001105078.2 | 227-208 | 58°C |
| EVI1_ex3L-F | CAGAGTTGAATCCTGCCTGGAATTCTCCAG | CB241806 | 243-275 | 56°C |
| EVI1_ex3L-R | GTATGGGAGCACCCTTCTGA | CB241806 | 462-443 | 56°C |
| RARβ_RARE-F | AAGCTCTGTGAGAATCCTG | DA403684 | 120-137 | 56°C |
| RARβ_RARE-R | GGATCCTACCCCGACGGTG | DA403684 | 406-388 | 56°C |

**Table S1: Primers used for RTQ-RT-PCR, RT-PCR, genomic PCR, and ChIP PCR.**

*Primer names. Forward primers are labeled with the suffix -F and reverse primers with the suffix -R. cEVI1 designates the sum of all EVI1 mRNA 5'-end variants. EVI1_1a, EVI1_1b, EVI1_1d, and EVI1_3L designate EVI1 5'-end variants starting with exons 1a, 1b, 1d, and 3L, respectively. cycD (*cyclophilinD*) served as an internal reference gene to control for the amount of cDNA present in each sample. EVI1_1a-F and EVI1_1a-R were used for RTQ-RT-PCR, while EVI1_1a-fwd and EVI1_1a-rev were used for conventional RT-PCR. Similarly, EVI1_3L-F and EVI1_3L-R were used for RTQ-RT-PCR, while EVI1_ex3L-F and EVI1_ex3L-R were used as a negative control for ChIP PCR. †Primer sequences are given from the 5'- to the 3'-end. EVI1_RARE-F and EVI1_RARE-R contained unrelated sequences at their 5'-ends, which are separated from the sequences matching the EVI1 sequence by an underscore. ‡Genbank accession numbers of the sequences used for primer design. §Nucleotide positions of the oligonucleotides, from 5' to 3', within the sequences indicated in the previous column. ¶AT, annealing temperature.

**SUPPLEMENTARY FIGURE**

Fig. S1: Expression and nuclear location of protein products derived from *EVI1* deletion constructs.

A) Schematic of the *EVI1* deletion constructs (as in Fig. 4A; replicated for reasons of clarity). Black boxes, zinc finger motifs; ZF1, ZF2, zinc finger domains 1 and 2, respectively; IR, intervening region; RD, repression domain; AR, acidic region. Amino acid positions delimiting these domains are indicated. All constructs are based on the pEFzeo vector backbone, and contain an N-terminal HA epitope tag. The SV40 large T antigen nuclear localization signal (NLS) was engineered onto the ZF1 construct; all other constructs include a predicted NLS contained in the IR. B) Immunoblot analysis. Protein extracts prepared from NTERA-2 cells transiently transfected with the indicated *EVI1* deletion constructs were subjected to immunoblot analysis using an HA antibody. Sizes of the bands of a molecular weight marker (MW) are shown to the left. C) Immunofluorescence analysis. NTERA-2 cells transiently transfected with the indicated *EVI1* deletion constructs were fixed and stained with an HA antibody and a FITC-conjugated secondary antibody. As a control, the location of endogenous EVI1 protein in untransfected NTERA-2 cells treated with DMSO or ATRA for 72 h was shown using an EVI1 antibody and an Alexa 468-conjugated secondary antibody. DNA was counterstained with DAPI. Images were recorded at 400-fold magnification.

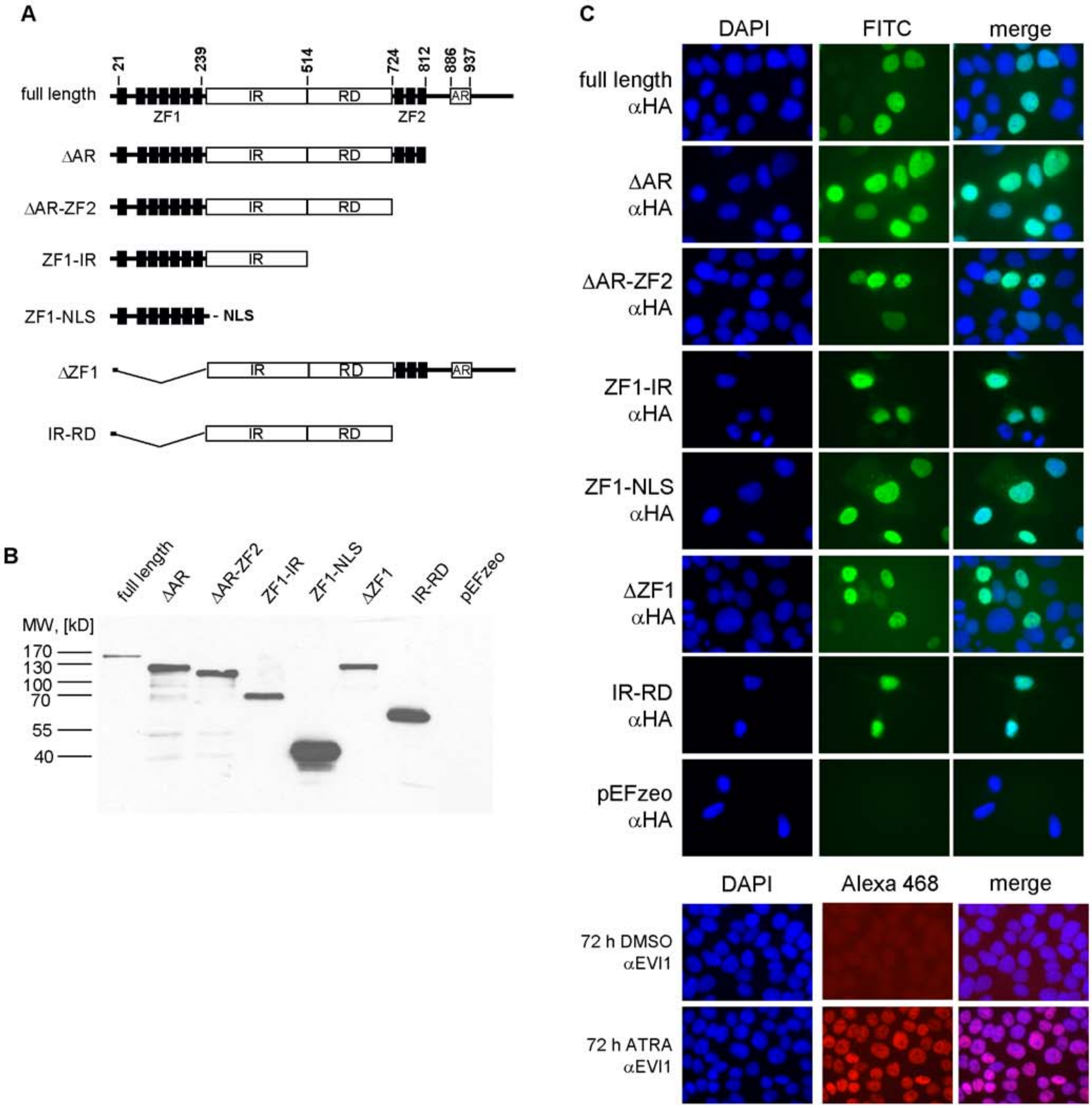

A
21
239
514
724
812
886
937
full length
IR
RD
AR
ZF1
ZF2
ΔAR
ΔAR-ZF2
ZF1-IR
ZF1-NLS
- NLS
ΔZF1
IR-RD
B
full length
ΔAR
ΔAR-ZF2
ZF1-IR
ZF1-NLS
ΔZF1
IR-RD
pEFzeo
MW, [kD]
170
130
100
70
55
40
C
DAPI
FITC
merge
full length αHA
ΔAR αHA
ΔAR-ZF2 αHA
ZF1-IR αHA
ZF1-NLS αHA
ΔZF1 αHA
IR-RD αHA
pEFzeo αHA
DAPI
Alexa 468
merge
72 h DMSO αEVI1
72 h ATRA αEVI1

## SUPPLEMENTARY METHOD

### Immunofluorescence analysis

To determine the subcellular location of the protein products derived from the *EVI1* deletion constructs, NTERA-2 cells were seeded onto coverslips and transiently transfected with the respective plasmids. Two days later, cells were fixed with methanol and incubated with an anti-HA antibody (mouse anti-HA.11 clone 16B12, Covance; 1:200), followed by a FITC conjugated goat anti-mouse secondary antibody (Fluorescein-conjugated AffiniPure Goat-anti-Mouse IgG (H+L), Jackson Immunoresearch; 1:200). DNA was counterstained with 4',6'-diamidino-2-phenylindole dihydrochloride (DAPI; Roche). Images were recorded at 400-fold magnification using an Axioplan microscope and an Axiocam HSm camera supported by Axio Vision software (Zeiss). To determine the location of endogenous EVI1 protein, untransfected NTERA-2 cells were treated with ATRA for 0 or 72 h, fixed, and incubated with an antibody against EVI1 (rabbit anti-EVI1 C50E12, Cell Signaling Technology; 1:200), followed by an Alexa 468 conjugated goat anti-rabbit secondary antibody (Molecular probes; 1:750). DNA was counterstained with DAPI and images were recorded as described above.
